# Ccr4-Not Regulates RNA Polymerase I Transcription and Couples Nutrient Signaling to the Control of Ribosomal RNA Biogenesis

**DOI:** 10.1371/journal.pgen.1005113

**Published:** 2015-03-27

**Authors:** R. Nicholas Laribee, Amira Hosni-Ahmed, Jason J. Workman, Hongfeng Chen

**Affiliations:** University of Tennessee Health Science Center Department of Pathology and Laboratory Medicine and the UT Center for Cancer Research, Memphis, Tennessee, United States of America; Indiana University, Howard Hughes Medical Institute, United States of America

## Abstract

Ribosomal RNA synthesis is controlled by nutrient signaling through the mechanistic target of rapamycin complex 1 (mTORC1) pathway. mTORC1 regulates ribosomal RNA expression by affecting RNA Polymerase I (Pol I)-dependent transcription of the ribosomal DNA (rDNA) but the mechanisms involved remain obscure. This study provides evidence that the Ccr4-Not complex, which regulates RNA Polymerase II (Pol II) transcription, also functions downstream of mTORC1 to control Pol I activity. Ccr4-Not localizes to the rDNA and physically associates with the Pol I holoenzyme while Ccr4-Not disruption perturbs rDNA binding of multiple Pol I transcriptional regulators including core factor, the high mobility group protein Hmo1, and the SSU processome. Under nutrient rich conditions, Ccr4-Not suppresses Pol I initiation by regulating interactions with the essential transcription factor Rrn3. Additionally, Ccr4-Not disruption prevents reduced Pol I transcription when mTORC1 is inhibited suggesting Ccr4-Not bridges mTORC1 signaling with Pol I regulation. Analysis of the non-essential Pol I subunits demonstrated that the A34.5 subunit promotes, while the A12.2 and A14 subunits repress, Ccr4-Not interactions with Pol I. Furthermore, *ccr4Δ* is synthetically sick when paired with *rpa12Δ* and the double mutant has enhanced sensitivity to transcription elongation inhibition suggesting that Ccr4-Not functions to promote Pol I elongation. Intriguingly, while low concentrations of mTORC1 inhibitors completely inhibit growth of *ccr4Δ*, a *ccr4Δ rpa12Δ* rescues this growth defect suggesting that the sensitivity of Ccr4-Not mutants to mTORC1 inhibition is at least partially due to Pol I deregulation. Collectively, these data demonstrate a novel role for Ccr4-Not in Pol I transcriptional regulation that is required for bridging mTORC1 signaling to ribosomal RNA synthesis.

## Author Summary

All cells communicate their environmental nutrient status to the gene expression machinery so that transcription occurs in proportion to the nutrients available to support cell growth and proliferation. mTORC1 signaling, which is essential for this process, regulates Pol I-dependent rRNA expression. We provide evidence that the RNA polymerase II regulatory complex, Ccr4-Not, also is a novel Pol I regulator required for mTORC1-dependent control of Pol I activity. Ccr4-Not disruption increases Pol I transcription due to an inability to decrease Pol I interactions with the transcription factor Rrn3 when mTORC1 signaling is reduced. Additionally, genetic and biochemical evidence supports a role for Ccr4-Not as a positive regulator of Pol I transcription elongation as well. Surprisingly, while Ccr4-Not mutations profoundly inhibit growth when mTORC1 activity is reduced, this phenotype is reversed by simultaneously impairing Pol I transcription. Overall, our data demonstrate that the evolutionarily conserved Ccr4-Not complex mediates environmental signaling through mTORC1 to control Pol I transcription initiation and, additionally, to regulate Pol I elongation. These studies further suggest that uncoupling Pol I from upstream mTORC1 activity by targeting Ccr4-Not sensitizes cells to mTORC1 inhibitors which is a concept that could have implications for anti-cancer drug development.

## Introduction

Eukaryotic cells alter gene expression programs in response to changes in their environment, including nutrient availability and the presence of stress, by transmitting this information through nutrient-responsive signaling cascades to the transcriptional machinery [1]. This process is critically important for regulating rDNA transcription and ribosomal RNA (rRNA) biogenesis. Over 60% of cellular transcription in rapidly growing cells is mediated by RNA polymerase I (Pol I), the sole RNA polymerase responsible for the production of three (the 18S, 5.8S, and 25S in budding yeast) of the four rRNAs [2]. Transcription of the 5S rRNA, tRNAs and specific snRNA and snoRNAs is mediated by RNA polymerase III (Pol III), while RNA polymerase II (Pol II) transcribes all ribosomal protein (RP) genes and the ribosome biogenesis (Ribi) genes coding for the ancillary factors necessary to produce and assemble ribosomes [3]. Coordinating ribosomal transcription by these three distinct polymerases to produce ribosomal components in the appropriate stochiometries, and in proportion to nutrient availability, is critical. Dysregulation of this process may result in the formation of partial or non-functional ribosomes that could have deleterious effects on cell fitness. Promoting ribosomal biogenesis in nutrient poor environments may also suppress the ability of cells to enter into survival states, such as autophagy, which could reduce viability [3].

The yeast rDNA exists as a multicopy array on chromosome XII with the individual 35S and 5S rRNA genes organized such that they are divergently transcribed and separated by non-transcribed sequences with only approximately half of the ~100–200 rDNA repeats expressed in a given cell [3]. The 35S rDNA is transcribed by Pol I as a polycistronic RNA transcript consisting of the 5´ external transcribed sequence (ETS1), the 18S, the internally transcribed sequence 1 (ITS1), the 5.8S, the internally transcribed sequence 2 (ITS2), the 25S, and the 3´ external transcribed sequence (ETS2) [3,4]. This polycistronic RNA is co-transcriptionally and post-transcriptionally processed through complex endonucleolytic and exonucleolytic mechanisms to form the mature 18S, 5.8S and 25S rRNAs that assemble with ribosomal proteins to form functional ribosomes [3,5,6]. The factors necessary to achieve Pol I-specific transcription initiation at the rDNA promoter have been extensively characterized in yeast. These factors include the UAF complex (consisting of Rrn5, Rrn9, Rrn10, Uaf30, and histones H3 and H4), TATA-binding protein (TBP), and core factor (composed of Rrn6, Rrn7, and Rrn11) (reviewed in [4]). UAF, TBP, and core factor bind within the rDNA promoter to nucleate Pol I recruitment and initiate transcription. Additionally, the high mobility group (HMG) protein, Hmo1, binds to both the promoter and the rDNA gene body to create a specialized chromatin state devoid of canonical nucleosomal structure that facilitates Pol I transcription [7–9].

Another essential Pol I regulator is the transcription factor Rrn3 (TIF-IA/Rrn3 in mammals) which is one of the few Pol I transcriptional regulators conserved at the amino acid level from yeast to mammals [10,11]. Rrn3 interacts with phosphorylated Pol I holoenzyme, via the A43 subunit, to form transcriptionally-competent Pol I initiation complexes [12–14]. Approximately 2% of Pol I is associated with Rrn3 to form initiation complexes suggesting that control of Rrn3-Pol I interactions is rate-limiting for Pol I transcription initiation [13]. Rrn3-Pol I interactions occur specifically at the rDNA promoter where Rrn3 directly contacts the Rrn6 subunit of core factor, and as Pol I begins to elongate it dissociates from Rrn3 [14,15]. A particularly important signaling input controlling Rrn3-Pol I dynamics is relayed through mTORC1 which is crucial for coordinating Pol I transcription with environmental nutrient status [16,17]. The pharmacological agent rapamycin selectively inhibits mTORC1 to suppress anabolic and cell proliferative processes, including rRNA synthesis. In mammals, rapamycin treatment induces hypophosphorylation of Rrn3 serine 44 and hyperphosphorylation of serine 199 to block Rrn3-Pol I complex formation and rapidly decrease rRNA synthesis [18]. While yeast Rrn3 shares conserved serine residues with mammalian Rrn3 that are important for Rrn3 function, whether mTORC1-regulated phosphorylation of these serines occurs in vivo is unclear [19]. Recent studies in yeast have demonstrated that decreased mTORC1 signaling disrupts Rrn3-Pol I interactions through an alternative mechanism that involves reduced *RRN3* mRNA translation [16]. Decreased translation rapidly lowers Rrn3 protein expression since Rrn3 is constitutively ubiquitylated and degraded via the proteasome [16]. Although *RRN3* is an essential gene required for Pol I transcription, this requirement can be bypassed if a chimera construct expressing Rrn3 fused to the A43 Pol I subunit is substituted [20]. Cells expressing Rrn3-A43 fusions grow normally in nutrient rich media; however, they are hypersensitive to mTORC1 inhibition [20,21]. These studies highlight the critical importance for appropriate mTORC1-dependent Pol I regulation when cells are exposed to conditions of nutrient stress.

While Pol I transcription initiation mechanisms are well defined, those affecting Pol I elongation remain are unclear. Studies have demonstrated that integral components of the Pol I holoenzyme, including the A49, A34.5, A12.2, and A14 subunits (all of which are non-essential genes in yeast), are linked to Pol I transcription elongation [22–25]. Mutations within Pol I that affect transcription elongation also impair rRNA processing and lead to the generation of aberrant rRNA intermediates [26]. The mechanisms by which altered elongation causes these rRNA processing defects are not currently understood, but they are likely to be complex since ~70% of nascent rRNA undergoes significant co-transcriptional processing [6,27]. In particular, the small subunit (SSU) processome complex binds the rDNA to both stimulate Pol I transcription and co-transcriptionally cleave the nascent rRNA within ITS1 yet how the elongating Pol I complex interacts with SSU processome to properly process the nascent transcript is not understood [5,6]. Control of Pol II elongation is well-defined and a number of accessory factors act in this process by either stimulating the intrinsic elongation activity of Pol II or providing chromatin-modifying activities necessary for Pol II to transcribe through chromatin [28]. Recent studies have demonstrated many of these Pol II elongation factors also regulate Pol I elongation, yet their importance to rRNA biogenesis is only now being elucidated. Examples of such factors include the PAF complex, Spt4 and Spt5, the FACT histone chaperone, the chromatin remodeling enzymes SWI/SNF and Chd1, and the proteasome [29–34].

The Ccr4-Not complex is composed of nine distinct subunits, including the Ccr4 mRNA deadenylase, Caf1, Caf40, Caf130, and Not1-5, and has both cytoplasmic and nuclear functions important for cell growth and proliferation. In the cytoplasm, Ccr4-Not acts to degrade mRNAs via the Ccr4 subunit which is the main deadenylase activity in yeast [35]. In addition to this intrinsic deadenylase activity, the Not4 subunit contains a functional RING domain that utilizes either the Ubc4 or Ubc5 E2 enzymes to mediate ubiquitin transfer to a small number of substrates [35]. Not4-dependent substrate ubiquitylation results in either proteasome-dependent degradation or altered substrate function [36–38]. Ccr4-Not also interacts with the proteasome and Not4 loss causes defects in both proteasome integrity and function. These defects lead to accumulation of polyubiquitylated proteins and decreased free ubiquitin which is a phenotype not shared with *ccr4Δ* [38,39]. Nuclear Ccr4-Not regulates Pol II transcription at both the initiation and elongation phases of the transcription cycle and is functionally connected to the mRNA export pathway as well [35,40]. How Ccr4-Not regulates Pol II transcription is only now beginning to be defined, particularly for the elongation phase of the transcription cycle. When Pol II transcription elongation reactions are assembled in vitro, Ccr4-Not directly stimulates stalled Pol II elongation complexes suggesting it acts directly to regulate Pol II elongation [40]. Furthermore, Ccr4-Not mutants have Pol II elongation defects in vivo [40–42]. Whether Ccr4-Not also contributes to transcriptional regulation by other RNA polymerases has not been addressed. In this study, we provide evidence that supports a novel role for Ccr4-Not as a Pol I transcriptional regulator that controls both Pol I initiation and elongation. Furthermore, we demonstrate that a functional Ccr4-Not is required to decrease Pol I transcription upon mTORC1 inhibition, suggesting that this complex integrates upstream nutrient signaling through mTORC1 to downstream control of rRNA biogenesis.

## Results

### Ccr4-Not interacts with RNA Pol I and acts to limit rRNA synthesis under nutrient rich conditions

**Fig 1:**
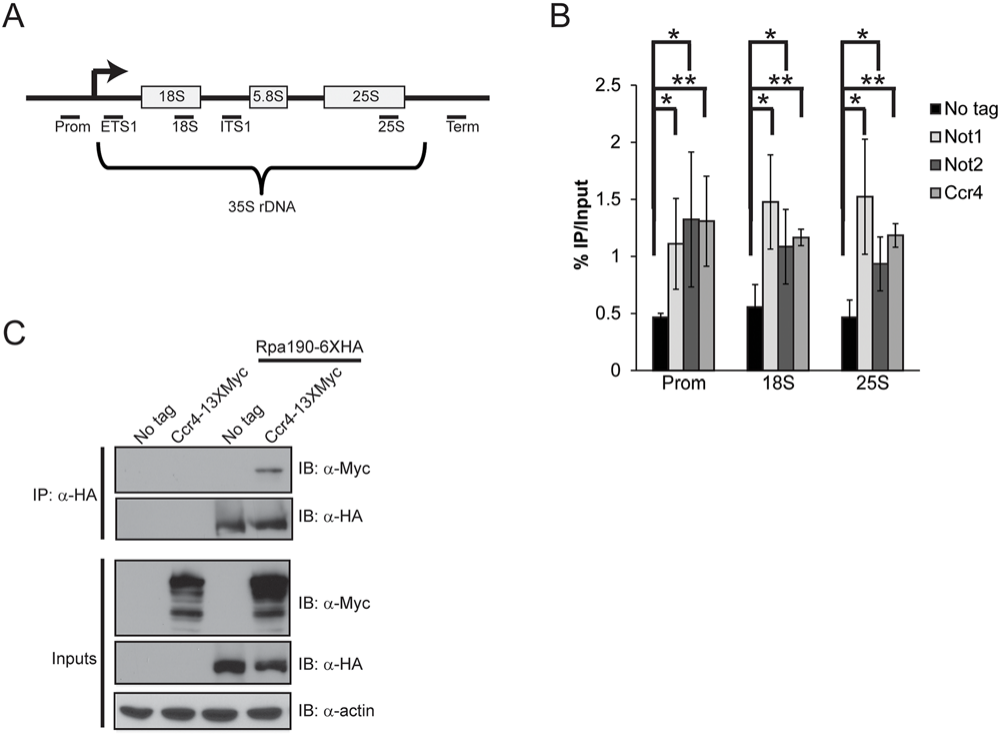
Ccr4-Not binds the rDNA and interacts with RNA Pol I. (A) Schematic of the rDNA locus. The relative position of the qPCR primers used are shown below as black bars. (B) ChIP of Ccr4-TAP, Not1-TAP, and Not2-TAP at the rDNA. The results are the average and standard deviation (SD) of three to four independent experiments. (C) Ccr4 associates with Pol I. 500 μg of cell extract from the indicated strains was utilized for α-HA immunoprecipitation and the presence of Ccr4 determined by α-Myc immunoblot. Statistical significance was determined by Student’s t-test. *- *p< 0*.*05*; **- *p<0*.*01*.

Specific Ccr4-Not mutants result in significant growth defects which prompted us to ask whether Ccr4-Not might contribute to Pol I transcription since rRNA biogenesis is rate limiting for cell growth and proliferation [35]. Utilizing yeast strains carrying C-terminal TAP epitope tags integrated at either the *CCR4*, *NOT1*, or *NOT2* genomic loci, we cultured cells in nutrient rich media (YPD) and performed chromatin immunoprecipitation (ChIP) coupled with quantitative PCR utilizing primers to the rDNA locus as outlined (Fig. 1A). All three Ccr4-Not subunits were significantly enriched above background suggesting that Ccr4-Not physically localizes to rDNA repeats (Fig. 1B) [43]. Next, we engineered strains to express either Rpa190-6XHA (Pol I catalytic subunit), Ccr4-13XMyc, or both, and performed co-immunoprecipitation (co-IP) experiments to determine if Ccr4-Not associates with Pol I. Precipitation of Pol I readily co-precipitated Ccr4, suggesting these complexes physically interact (Fig. 1C). Collectively, these data are consistent with the possibility that Ccr4-Not directly regulates Pol I transcription.

**Fig 2:**
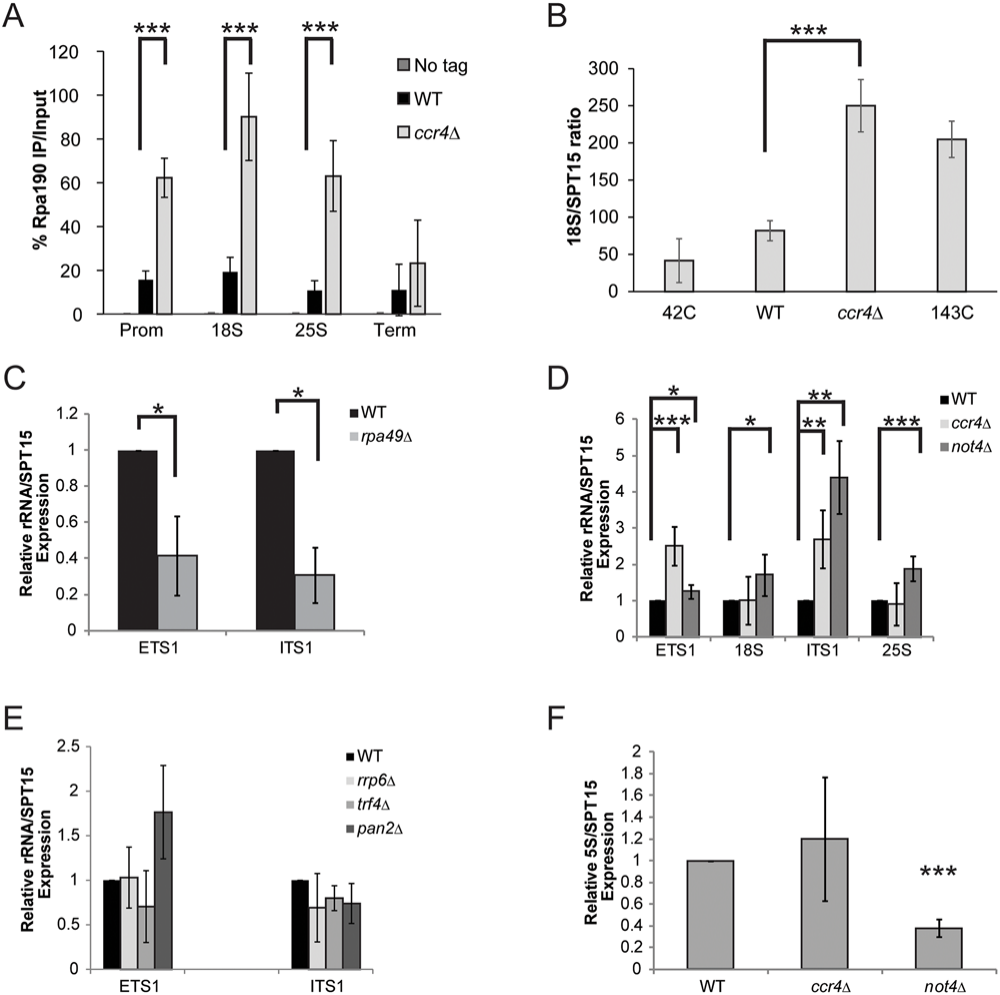
Ccr4-Not disruption increases Pol I transcription. (A) ChIP for the Pol I catalytic subunit (Rpa190-6XHA) in the no tag, wild-type (WT) and *ccr4Δ* backgrounds. The average and SD of five independent experiments are shown. (B) 18S/SPT15 ratio from strains 42C (NOY886), 143C (NOY1051), wild-type and *ccr4Δ*. The average and SD of three independent experiments are presented. (C) ETS1 and ITS1 rRNA expression in wild-type and *rpa49Δ*. Data are the average and SD of three independent experiments. (D) Expression of the indicated rRNA sequences in WT, *ccr4 Δ*, and *not4 Δ*. Data represent the average and SD of five independent experiments. (E) ETS1 and ITS1 rRNA expression from wild-type and the indicated RNA processing mutants. The average and SD of four experiments are shown. (F) Level of 5S rRNA in WT, *ccr4 Δ*, and *not4 Δ*. Data are the average and SD of five independent experiments. Student’s t-test determined statistical significance. *- *p< 0*.*05*; **-*p< 0*.*01*; ***- *p< 0*.*005*.

To address the role of Ccr4-Not in Pol I transcription, we performed Pol I-specific ChIP experiments in wild-type and *ccr4Δ* cells grown to log phase in YPD. Surprisingly, under these conditions we detected significantly elevated levels of Pol I throughout the rDNA suggesting Ccr4-Not normally acts to limit Pol I rDNA binding (Fig. 2A). Deficiencies in Pol I regulators can also influence rDNA copy number so we next determined whether *ccr4Δ* resulted in significant changes to rDNA copy number. Utilizing our ChIP samples, and ChIP samples from yeast strains containing either 42 rDNA copies (42C) or 143 copies (143C) [44], we performed quantitative PCR with primers to the 18S and also to the essential single-copy gene, *SPT15*, to generate an 18S/SPT15 ratio. As expected, analysis of the 42C strain gave a low ratio whereas the analysis of the 143C strain resulted in a higher ratio (Fig. 2B). Interestingly, the *ccr4Δ* resulted in an 18S/SPT15 ratio higher than that detected in wild-type and the 143C strain (Fig. 2B). Because our Pol I ChIP signals are normalized to input DNA, this increase in rDNA copy number is accounted for in the analysis and thus cannot explain the increased Pol I ChIP signal. Therefore, these data suggest that *ccr4Δ* causes increased Pol I transcription as well as an increase in rDNA copy number.

The increased Pol I bound to the rDNA suggested the possibility that *ccr4 Δ* increases overall rRNA transcription. Nascent rRNA synthesis is typically measured through incorporation of radiolabelled methionine or uridine via pulse-chase experiments of cells grown in nutrient defined media. However, both Pol I transcriptional activity, as well as the biological functions of Ccr4-Not, are downstream targets of nutrient regulated signaling pathways that may be differentially affected by growth under nutrient rich versus nutrient defined conditions [17,45,46]. Therefore, measuring rRNA synthesis via this approach could mask biological effects that occurring during growth in nutrient rich conditions (see below). Therefore, we elected to quantify differences in nascent rRNA levels by measuring the expression of ETS1 and ITS1. These sequences exist transiently in the nascent rRNA since ~70% of nascent rRNA is co-transcriptionally processed in these regions, and their presence has been utilized previously as a general proxy for Pol I transcriptional activity [27,47,48]. Initially, we confirmed that we could detect significant alterations in nascent rRNA levels via qPCR by synthesizing randomly-primed cDNA from total RNA isolated from wild-type and *rpa49 Δ* mutant cells. These samples were analyzed with primers specific to either ETS1 or ITS1 and normalized to the expression of the *SPT15* housekeeping gene. *RPA49* encodes the non-essential Pol I A49 subunit whose loss significantly impairs Pol I transcription [25]. Under these conditions, significant reductions in both ETS1 and ITS1 were detected in *rpa49 Δ* compared to wild-type (Fig. 2C) suggesting this approach could be utilized to detect significant perturbations in nascent rRNA synthesis for cells grown under nutrient rich conditions.

Utilizing this approach, we determined the effect that loss of either enzymatic activity associated with Ccr4-Not, specifically the Ccr4 deadenylase or Not4 ubiquitin ligase subunits, had on rRNA synthesis. Intriguingly, both *ccr4 Δ* and *not4 Δ* resulted in elevated expression of ETS1 and ITS1 relative to wild-type (Fig. 2D). Additionally, the *not4 Δ* also exhibited a subtle, but reproducibly significant, increase in 18S and 25S rRNA levels as well (Fig. 2D). Since this approach does not delineate between differences in nascent 18S and 25S rRNA synthesis versus changes in their overall stability, how *not4 Δ* increases 18S and 25S rRNA expression is currently unknown. Defects in Pol I transcription elongation generate aberrantly processed rRNA intermediates that are subsequently cleared through the collective actions of both the TRAMP polyadenylation complex and the nuclear exosome. As a consequence, mutations in TRAMP or exosome can result in accumulation of these non-processed rRNA intermediates [26,49]. Ccr4-Not interacts with both TRAMP and the exosome and has been suggested to regulate the functions of both complexes [50]. Therefore, the elevated ETS1 and ITS1 expression in *ccr4 Δ* or *not4 Δ* could be explained either by increased Pol I transcription or by the enhanced stability of these nascent sequences due to TRAMP and/or exosome defects. To rule out this latter possibility, we analyzed ETS1 and ITS1 expression from wild-type, *trf4 Δ* (a TRAMP mutant), and *rrp6 Δ* (nuclear exosome mutant) backgrounds. Additionally, we also examined ETS1 and ITS1 expression in a catalytic mutant (*pan2 Δ*) of the Pan2-Pan3 complex which is an additional mRNA deadenylase complex that is partially redundant with Ccr4-Not [51]. None of these mutants significantly increased expression of either ETS1 or ITS1 compared to wild-type (Fig. 2E). These data demonstrate that the effect Ccr4-Not disruption has on rRNA synthesis is specific to Ccr4-Not and is not due to a generalized disruption in rRNA processing. Instead, when combined with the ChIP data from above, the results are most consistent with Ccr4-Not mutation causing an increase in Pol I-dependent rRNA synthesis. The expression of 5S rRNA, which is transcribed by Pol III, was next assessed to determine the extent of the role Ccr4-Not has in rRNA biogenesis. Although *ccr4 Δ* did not affect 5S expression, *not4Δ* severely impaired 5S rRNA levels (Fig. 2F). Collectively, these data implicate Ccr4-Not as a negative regulator of 35S synthesis while the Not4 subunit appears to also have a positive role in 5S rRNA expression. As *not4 Δ* is considerably more growth impaired than *ccr4Δ* and has additional effects that might confound our analysis, we chose to focus predominantly on the role by which the Ccr4 subunit contributes to Pol I transcription.

### Loss of Ccr4 alters binding of select Pol I transcriptional regulators

**Fig 3:**
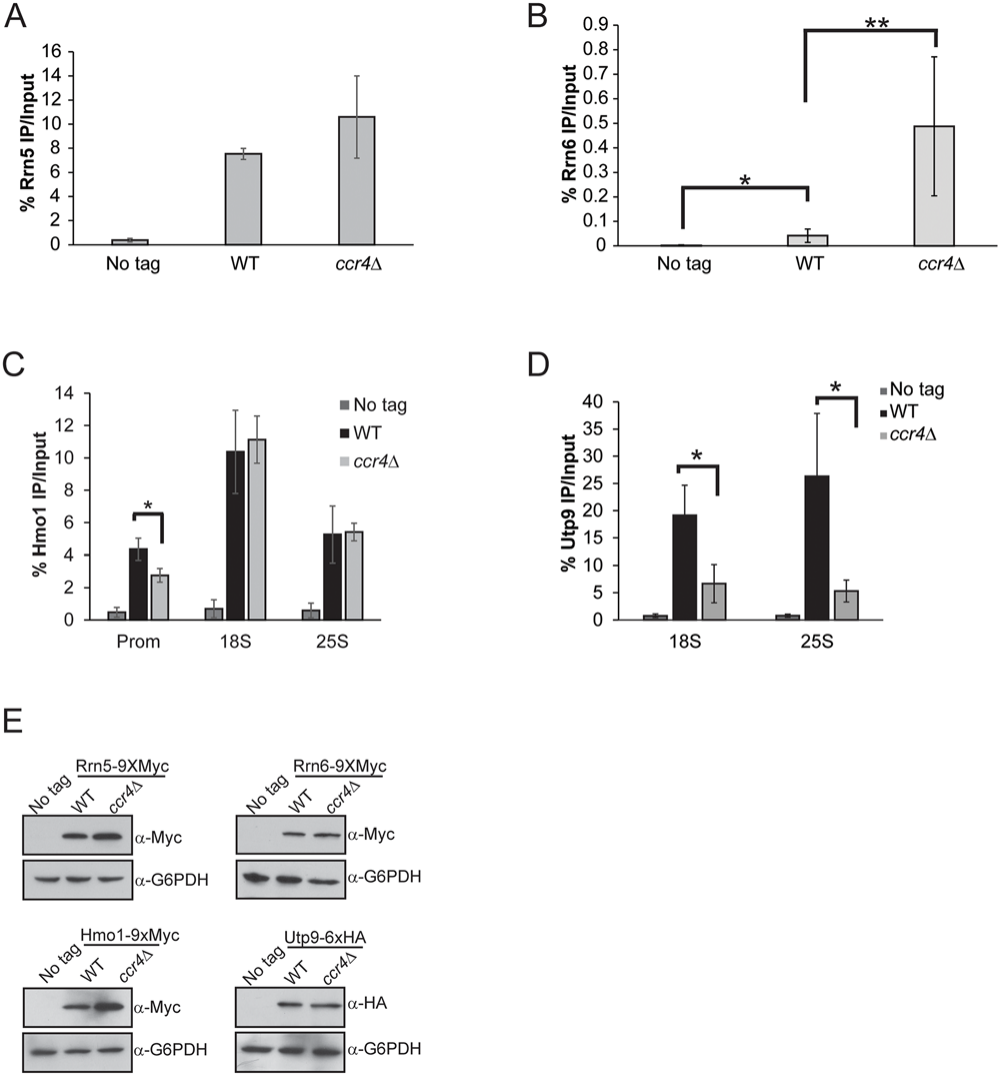
Ccr4-Not has selective effects on Pol I transcriptional regulators. (A) Promoter ChIP for the UAF subunit Rrn5. The average and SD of three independent experiments are reported. (B) Rrn6 binding to the rDNA promoter. Five independent experiments were performed and the average and SD are shown. (C) ChIP for Hmo1 binding along the rDNA gene body. Data are the average and SD of four independent experiments. (D) ChIP for the SSU processome subunit Utp9 in the rDNA gene body. The average and SD of three independent experiments are reported. (E) 30 μg of cell extracts were resolved by SDS-PAGE and the indicated immunoblots for factors in A-D were performed. Statistical significance was determined by Student’s t-test. *- *p< 0*.*05*; **- *p<0*.*01*

To further understand the mechanism by which *ccr4Δ* causes increased Pol I transcription, we next examined components of the Pol I transcription initiation machinery bound at the promoter. The UAF complex stably binds both transcriptionally active and inactive rDNA promoters and serves to facilitate high level Pol I transcription initiation [4,17,52]. We epitope tagged the *RRN5* (UAF subunit) locus with wild-type and *ccr4Δ* cells and then monitored Rrn5 promoter association by ChIP. Rrn5 binding was not significantly increased in *ccr4Δ* suggesting that Ccr4-Not disruption did not augment Pol I transcription by elevating UAF promoter interactions (Fig. 3A). Next, we genomically epitope-tagged the *RRN6* (core factor subunit) genomic locus in wild-type and *ccr4Δ* cells and monitored Rrn6 binding. In wild-type cells, we detected a modest, but statistically significant, enrichment of Rrn6 over background which is likely due to the very low expression levels of core factor in cells (Fig. 3B) [53]. Intriguingly, the *ccr4Δ* resulted in enhanced Rrn6 enrichment suggesting that *ccr4Δ* either increased core factor binding or that Rrn6 was made more accessible for immunoprecipitation in this mutant. This same experimental approach was then applied to the analysis of two additional Pol I transcriptional regulators, specifically the high mobility group protein Hmo1 and the SSU processome complex via the Utp9 subunit. These experiments demonstrated that *ccr4Δ* caused a modest decrease in Hmo1 binding that was restricted solely to the promoter (Fig. 3C) whereas the SSU processome was reduced throughout the transcribed rDNA (Fig. 3D). Since the SSU processome mediates co-transcriptional cleavage of the nascent rRNA within ITS1, reduced rDNA binding by this complex likely contributes to the increase in ITS1 levels identified in Ccr4-Not mutants (Fig. 2D) [5,6]. The differences in rDNA binding by these Pol I regulators cannot be explained by changes in their expression as *ccr4Δ* does not alter the protein levels of Rrn5, Rrn6 or Utp9 and it modestly increases expression of Hmo1 (Fig. 3E). Overall, these data demonstrate that *ccr4Δ* selectively affects Pol I transcriptional regulators and that the increase in core factor enrichment correlates well with the elevated Pol I transcription occurring in *ccr4Δ*.

### Ccr4-Not restricts initiation-competent Pol I complex formation which is function that is modified by the cellular nutrient environment

**Fig 4:**
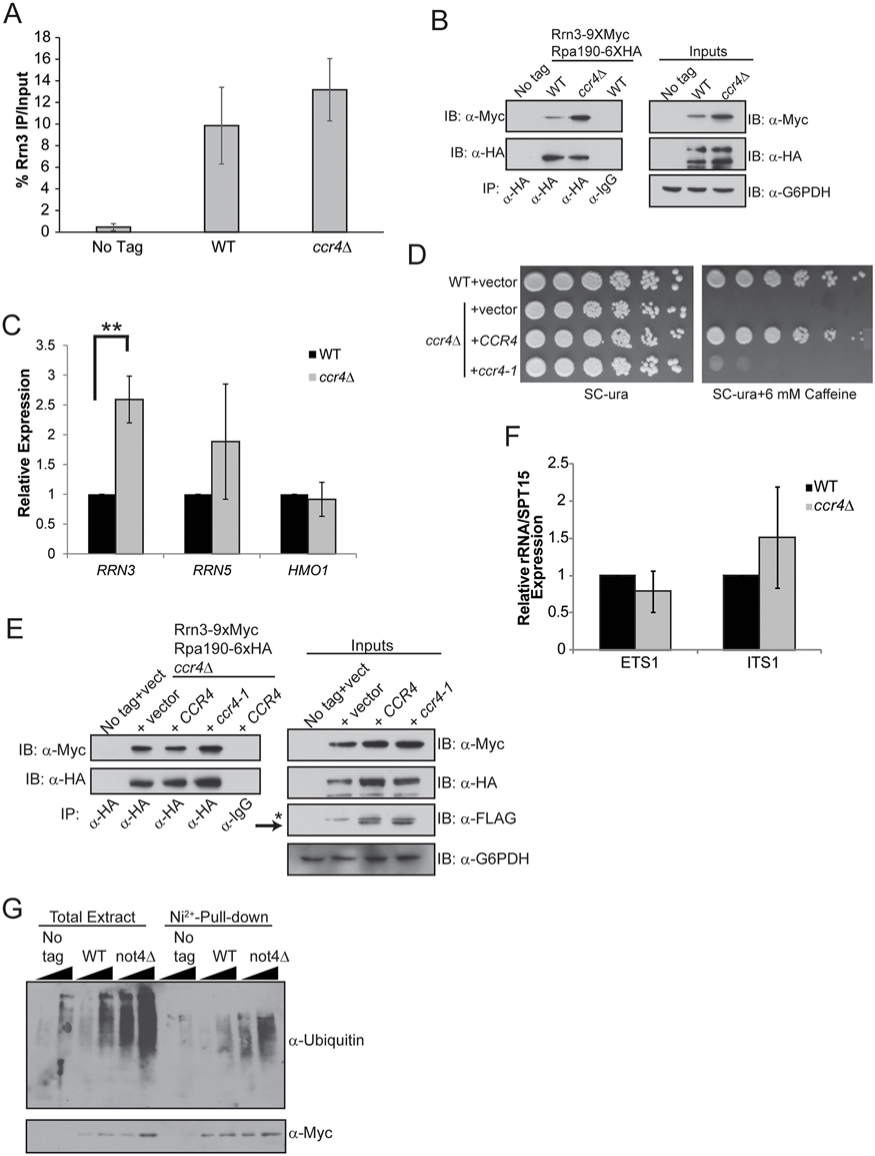
Loss of Ccr4-Not function disrupts regulation of Rrn3-Pol I complex formation. (A) ChIP for Rrn3 at the rDNA promoter. Three independent experiments were performed and the average and SD are reported. (B) Rrn3-Pol complex formation and basal Rrn3 protein levels are both increased from *ccr4Δ* cells cultured in nutrient rich media. α-HA immunoprecipitations were performed from no tag control, wild-type and *ccr4Δ* extracts. Samples were resolved by 8% SDS-PAGE and Rrn3 co-association determined by α-Myc immunoblot. Inputs represent 30 μg of cell extract. (C) Expression of the indicated genes was evaluated using the indicated gene-specific primers and then normalized to the expression of the *SPT15* housekeeping gene. Wild-type was set to a value of 1 for each gene and the expression in *ccr4Δ* was then expressed relative to wild-type. Data are the average and SD of four independent experiments. (D) Spotting assay for WT cells expressing control vector or *ccr4Δ* cells reconstituted either with control, *CCR4*, or *ccr4-1* expression vectors. The indicated strains were grown in SC-ura media overnight before equal numbers of cells were serially diluted 5-fold and spotted to the indicated plates. Plates were incubated for four days at 30°C. (E) Culturing WT and *ccr4Δ* cells in nutrient defined media (SC-ura) prevents increased Rrn3-Pol I complex formation. Cell extracts from strains cultured in nutrient defined (SC-ura) media were used in α-HA immunoprecipitations to measure Rrn3-Pol I complex formation. α-IgG is the negative control co-IP. Inputs represent 30 μg of cell extracts while the asterisk represents a residual signal that was not completely removed after stripping the previous immunoblot. Arrow indicates the Ccr4-FLAG specific bands. (F) WT and *ccr4Δ* cells without plasmids were cultured in nutrient defined (SC) media before harvesting total RNA and quantifying ETS1 and ITS1 expression. Average and SD of four independent experiments are presented. (G) A *not4Δ* results in accumulation of polyubiquitylated Rrn3. The Rrn3 pull-down was performed as described in the Methods. Duplicate sets of samples were resolved by 8% SDS-PAGE and one set was probed with α-ubiquitin and the other with 0α-Myc. Student’s t-test determined statistical significance. **-*p< 0*.*01*.

Pol I interaction with the transcription factor Rrn3 is essential for forming transcriptionally competent Pol I initiation complexes [12–14]. The Rrn6 subunit of core factor also contacts Rrn3 which contributes to Pol I transcription initiation by facilitating promoter recruitment of the Rrn3-Pol I initiation complex [14]. We next assessed if the increased Pol I transcription in *ccr4Δ* could be explained by changes in Rrn3-Pol I interactions. Wild-type and *ccr4Δ* cells expressing endogenous, genomically-tagged Rrn3-9XMyc and Rpa190-6XHA were grown in nutrient rich media and Rrn3 ChIP was performed. No difference in Rrn3 promoter binding was detected in *ccr4Δ* cells, demonstrating that Ccr4-Not disruption does not increase Rrn3 promoter association (Fig. 4A). However, the amount of Rrn3 expressed in *ccr4Δ*, as well as the amount of Rrn3-Pol I complexes formed, was significantly enhanced in *ccr4Δ* (Fig. 4B). Examination of mRNA expression for *RRN3*, as well as *RRN5* and *HMO1*, identified a selective, approximately 2.5-fold increase in *RRN3* expression in *ccr4Δ* (Fig. 4C). These data suggest that Ccr4-Not regulates either *RRN3* transcription or *RRN3* mRNA stability to limit the total amount of Rrn3 available to form transcriptionally competent Rrn3-Pol I complexes.

To further address the role of Ccr4-Not in Rrn3-Pol I complex formation, we transformed *ccr4Δ* cells with control vector or vectors overexpressing C-terminally FLAG-tagged wild-type *CCR4* or catalytically inactive mutant *ccr4-1*. We confirmed that expression of wild-type, but not mutant, Ccr4 rescued growth on caffeine since *ccr4Δ* cells are caffeine sensitive (Fig. 4D) [54]. Next, we cultured cells in nutrient defined (SC) media lacking uracil to select for plasmid maintenance and prepared whole cell extracts for α-Pol I precipitation. Surprisingly, and in contrast to what was detected when cells were grown in nutrient rich YPD, the Pol I precipitated under these conditions co-precipitated equivalent amounts of Rrn3 from cells transformed with control vector or vectors expressing wild-type or mutant Ccr4 (Fig. 4E). Rrn3 protein expression was comparable between these strains as well (Fig. 4E). This result was surprising since *ccr4Δ* cells cultured in YPD consistently expressed more Rrn3 protein and had greater Rrn3-Pol I complex formation (Fig. 4B). Because this experiment was performed using vector-transformed cells grown in SC-uracil media to maintain plasmid selection, we speculated that the nutrient environment might alter the effect *ccr4Δ* has on Pol I transcription. To test this possibility, we cultured wild-type and *ccr4Δ* in complete SC media and analyzed ETS1 and ITS1 expression. Under these conditions, *ccr4Δ* did not increase rRNA expression (Fig. 4F) which contrasts with the result from cells cultured in YPD (compare Fig. 4F and Fig. 2D). These data demonstrate that under defined, nutrient limiting conditions loss of Ccr4-Not function neither increases Rrn3-Pol I complex formation nor elevates rRNA synthesis.

Rrn3 is constitutively ubiquitylated and degraded via the proteasome [16]. A *not4Δ* impairs proteasome assembly and function which results in an accumulation of polyubiquitylated proteins [38,39]. We speculated that in *not4Δ*, polyubiquitylated Rrn3 may accumulate due to dysfunctional proteasome regulation and that this stabilized Rrn3 may contribute to the increased rRNA synthesis detected in the *not4Δ* background (Fig. 2D). To test this possibility, we integrated a 1XMyc/7X-histidine tag at the *RRN3* genomic locus in wild-type and *not4Δ* cells. Duplicate wild-type and *not4Δ* cultures, as well as the corresponding no tag control, were grown to log phase in YPD, lysed in denaturing buffer, and the Rrn3 from these samples was purified using nickel-conjugated agarose resin. The purified Rrn3 was then either immunoblotted with α-ubiquitin or α-Myc. In these experiments, the *not4 Δ* caused a notable increase in total cellular polyubiquitylated proteins relative to wild-type cells which is consistent with previous reports (Fig. 4G) [38,39]. In particular, *not4Δ* caused a pronounced increase in polyubiquitylated Rrn3 suggesting that the ubiquitylated protein was not being efficiently degraded in these cells (Fig. 4G). Since Rrn3 is still ubiquitylated in *not4Δ*, Not4 cannot be the sole E3 ligase responsible for Rrn3 ubiquitylation and subsequent proteasome-dependent turnover. However, these data do suggest that *not4Δ* loss stabilizes the pool of Rrn3 indirectly by disrupting proteasome function which prevents efficient Rrn3 degradation. Taken together, these data suggest Ccr4-Not negatively regulates Rrn3-Pol I complex formation and, consequently, Pol I transcription initiation. However, this role is influenced by the cellular nutrient environment as the increase in Rrn3-Pol I complex formation and elevated rRNA synthesis caused by *ccr4Δ* did not occur when the mutant was grown in nutrient defined media.

### Ccr4-Not disruption uncouples Pol I transcriptional regulation from upstream mTORC1 signaling

**Fig 5:**
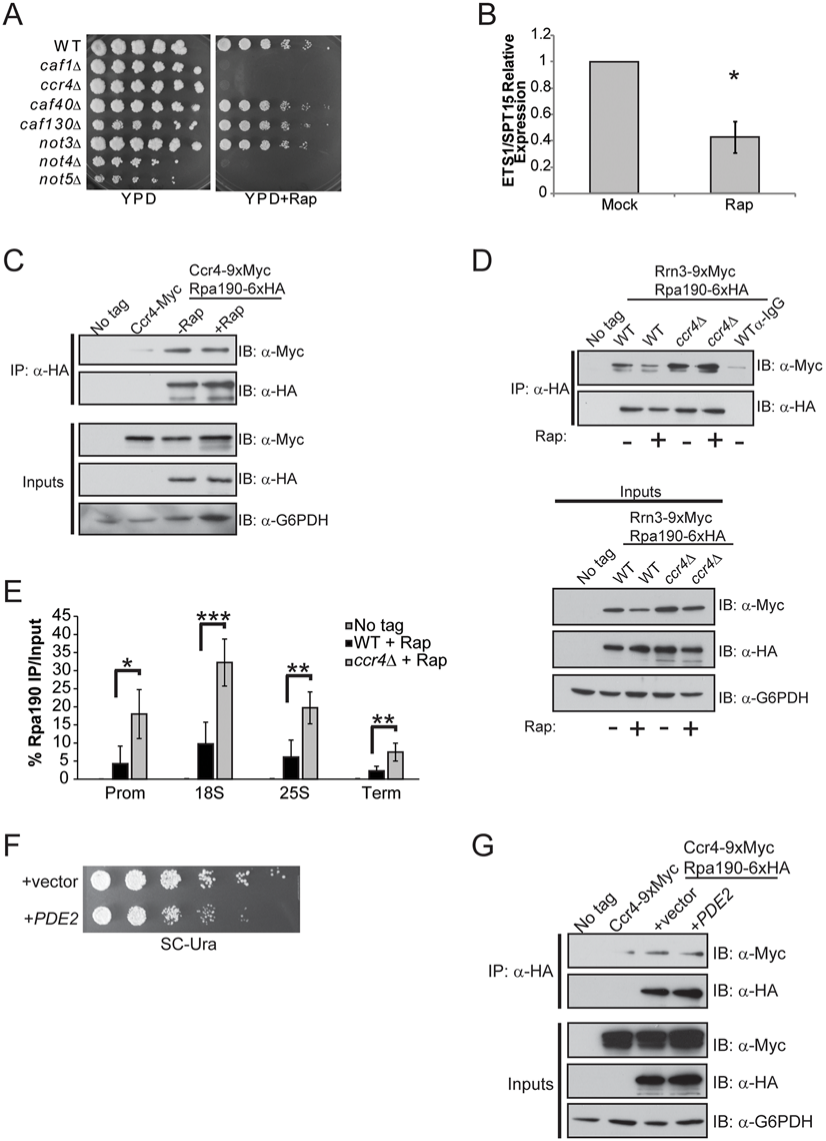
mTORC1 regulation of Rrn3-Pol initiation complex formation depends on functional Ccr4-Not. (A) Spotting assay of WT and the indicated Ccr4-Not deletion mutants on nutrient rich (YPD) or YPD + 25 nM rapamycin. Plates were incubated at 30°C for four days. (B) Expression of ETS1 in WT cells either mock-treated or treated with 200 nM rapamycin for 30 minutes. The average and SD of three independent experiments are shown. (C) Ccr4-Not association with Pol I is not disrupted when mTORC1 is inhibited. The indicated strains were cultured in YPD before mock treating or treating with 200 nM rapamycin for 30 minutes. Cell extracts were prepared, Pol I was immunoprecipitated and the association of Ccr4 determined by α-Myc immunoblot. Inputs represent 30 μg of total cell extract. (D) mTORC1 down regulation does not disrupt Rrn3-Pol complex formation in *ccr4Δ*. The indicated strains were cultured and the experiment was performed as in (C). α-IgG indicates negative control immunoprecipitation using extracts from the mock-treated WT sample. The data are representative of three independent experiments. (E) ChIP for Pol I in wild-type and *ccr4Δ* cells treated with rapamycin as in (D). Data are the average and SD of four independent experiments. (F) The Ccr4-9XMyc/Rpa190-6XHA strain was transformed with control vector or vector overexpressing *PDE2*. Cells were cultured overnight in SC-leu media and then equal numbers of cells were 5-fold serially diluted and spotted onto a SC-leu plate. The plate was incubated for four days at 30°C. (G) The indicated strains from (F) were cultured in SC-leu media before preparing cell extracts and performing Pol I immunoprecipitations as in (C). Statistical significance was determined by Student’s t-test. *- *p< 0*.*05*; **- *p<0*.*01*; ***- *p<0*.*005*

The differential effects on rRNA synthesis detected in *ccr4Δ* cells grown in YPD versus SC suggest nutrient signaling pathways may affect the role of Ccr4-Not in Pol I transcriptional control. The mTORC1 and Ras/PKA pathways are the two main signaling pathways that couple nutrient availability to cell growth control with mTORC1 specifically regulating Pol I transcription by controlling Rrn3-Pol I complex formation [1,16]. We initially addressed whether Ccr4-Not might participate in mTORC1-dependent Pol I regulation. Deletion of specific Ccr4-Not subunits (*ccr4Δ*, *caf1Δ*, *not4Δ* and *not5Δ*) causes profound rapamycin sensitivity, consistent with previous studies linking Ccr4-Not to the mTORC1 pathway (Fig. 5A) [55]. Next, we cultured wild-type cells in YPD in the absence or presence of an inhibitory (200 nM) concentration of the specific mTORC1 inhibitor rapamycin for 30 minutes to confirm suppression of Pol I transcription. mTORC1 inhibition resulted in a significant reduction in nascent rRNA synthesis by approximately 60% which is consistent with previous studies (Fig. 5B) [16,17]. Surprisingly, under these conditions Ccr4-Not association with Pol I is not disrupted even though mTORC1 inhibition was previously demonstrated to cause Pol I nucleolar delocalization (Fig. 5C) [46].

To determine the consequence mTORC1 inhibition has on Rrn3-Pol I interaction in wild-type and *ccr4Δ* cells, this experiment was repeated and the relative amounts of Rrn3-Pol I complexes were assessed. We detected fewer Rrn3-Pol I complexes in rapamycin-treated wild-type cells compared to mock-treated controls, a result which is consistent with the reduction in Rrn3 protein that occurs under these conditions (Fig. 5D) [16]. Surprisingly, mTORC1 inhibition in *ccr4Δ* did not significantly impair Rrn3-Pol I complex formation even though overall levels of Rrn3 were modestly decreased in the mTORC1-inhibited samples (Fig. 5D). More Pol I remained bound to the rDNA in *ccr4Δ* compared to wild-type under these conditions as well (Fig. 5E). To address whether Ras/PKA signaling may affect Ccr4-Not interactions with Pol I, we overexpressed the high affinity cyclic AMP (cAMP) phosphodiesterase, *PDE2*. Increased Pde2 expression facilitates cAMP breakdown, thus reducing PKA signaling and slowing cell growth and proliferation (Fig. 5F) [1]. Pol I immunoprecipitated from control and *PDE2* overexpressing cell extracts retained equivalent levels of associated Ccr4, demonstrating that Ccr4-Not interactions with Pol I are insensitive to upstream Ras/PKA activity and to conditions that result in generalized slow growth (Fig. 5G). In total, these data demonstrate that Ccr4-Not remains tightly associated with Pol I even under conditions that suppress the activity of nutrient signaling pathways controlling Pol I transcription (mTORC1) or regulate other aspects of cell growth and proliferation (Ras/PKA). Furthermore, Ccr4-Not disruption uncouples Pol I from mTORC1 control by both maintaining Rrn3-Pol I complex formation as well as retaining Pol I bound to the rDNA when mTORC1 signaling is suppressed.

### Pol I elongation subunits regulate Ccr4-Not association and mitigate sensitivity of Ccr4-Not disruption to mTORC1 inhibition

**Fig 6:**
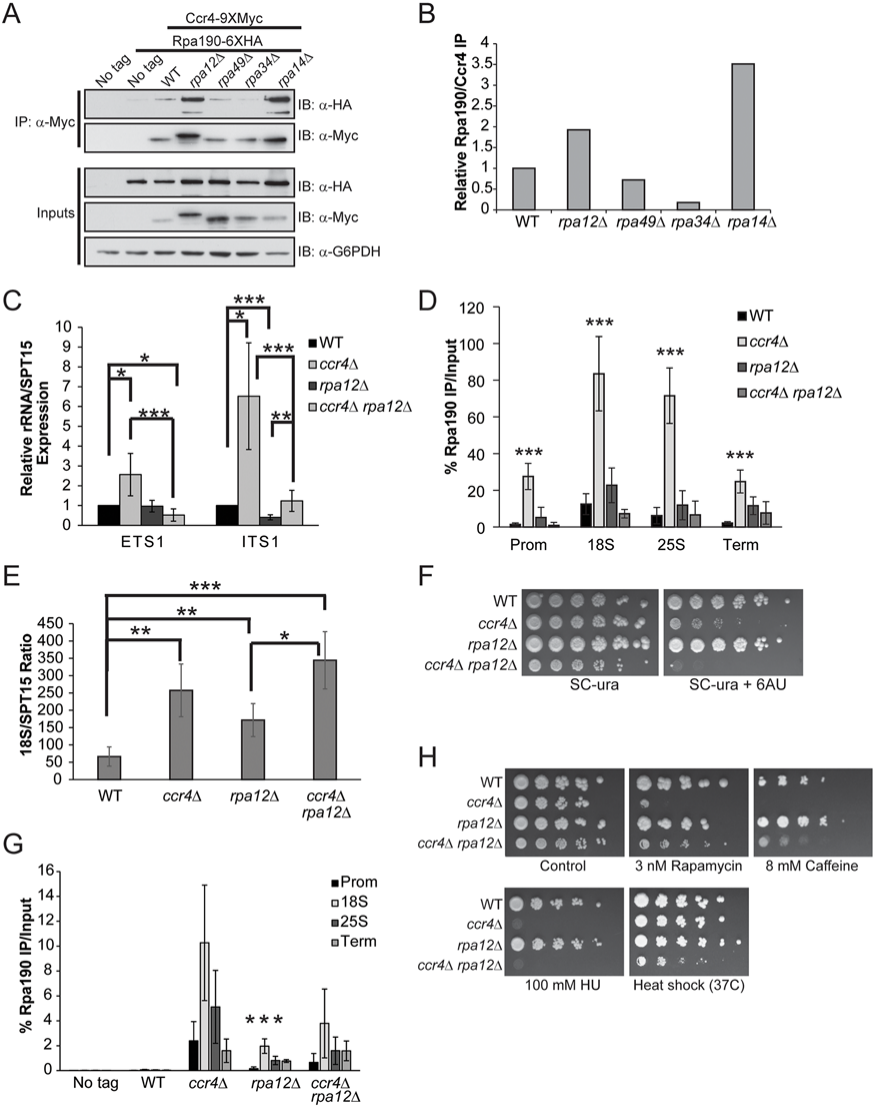
Pol I elongation subunits modulate Ccr4-Not interaction and the sensitivity of Ccr4-Not mutants to mTORC1 inhibition. (A) Pol I elongation mutants were generated in the Ccr4-9XMyc Rpa190-6XHA background and the amount of Pol I associated with Ccr4 determined by α-Myc immunoprecipitation. (B) Quantification of immunoblots from (A). The amount of Rpa190 co-immunoprecipitated was normalized to the total amount of Ccr4 present. WT was set to a value of 1 and the individual Pol I deletion mutants were expressed relative to WT. Data are representative of four independent experiments. (C) Expression of ETS1 and ITS1 was quantified in WT, *ccr4 Δ*, *rpa12Δ*, and *ccr4Δ rpa12Δ* cells cultured in nutrient rich media. Data are the average and SD of five independent experiments. (D) Duplicate experiments from (C) were utilized for Pol I ChIP. Data are the average and SD of four independent experiments. (E) The 18S/SPT15 ratio for the indicated strains was determined. The average and SD of four independent experiments are presented. (F) Sensitivity of Ccr4 and Rpa12 mutants to the transcription elongation inhibitor 6AU. Wild-type and the indicated individual or double mutant strains were spotted to SC-ura or SC-ura containing 15 μg/mL 6AU and incubated for six days. (G) Wild-type and the indicated mutants from (F) were cultured to log phase in nutrient rich media and treated with 15 μg/mL 6AU for 20 minutes before performing α-Pol I ChIP. Four independent experiments were performed and the average and SD plotted. Asterisks indicates a significant reduction of Pol I promoter binding in *rpa12Δ* relative to *ccr4Δ* (H) Impairing Pol I activity rescues sensitivity of *ccr4Δ* to mTORC1 inhibition. The indicated strains were cultured overnight in YPD and then equal numbers of cells were 5-fold serially diluted and spotted to control (YPD) plates or plates containing the indicated concentration of mTORC1 (rapamycin or caffeine) or DNA replication (hydroxyurea) inhibitor. Plates were incubated at 30°C or 37°C for four days. *- *p< 0*.*05*; **- *p< 0*.*01*; ***- *p< 0*.*005*. Student’s t-test determined statistical significance.

The data presented above suggests Ccr4-Not functions in Pol I transcription initiation by affecting core factor function and controlling the formation of Rrn3-Pol I complexes in a nutrient-dependent fashion. However, the recruitment of Ccr4-Not along the entire rDNA, its co-association with Pol I, and the fact that Ccr4-Not directly regulates Pol II transcription elongation all suggest it may function in Pol I elongation as well. To pursue this possibility, we determined if any of the four non-essential Pol I subunits were required for Ccr4-Not to interact with Pol I by engineering wild-type and individual Pol I deletion mutants to express Ccr4-9XMyc combined with Rpa190-6XHA. Ccr4-9XMyc was then immunoprecipitated from extracts prepared from these strains grown under nutrient rich conditions. Precipitation of Ccr4 specifically co-precipitated Pol I, consistent with our previous results (Fig. 6A and 1C). While deletion of the Pol I A49 subunit (*rpa49Δ*) did not alter Ccr4-Not co-association, deletion of the A34.5 (*rpa34Δ*) subunit reduced Ccr4-Not association to background levels (Fig. 6A and 6B). These results were unexpected since A49 binds A34.5 to form a heterodimeric subcomplex within Pol I, and A49 loss has been reported to release A34.5 from the remainder of Pol I [23,25]. Surprisingly, deletion of either the A12.2 (*rpa12Δ*) or the A14 (*rpa14Δ*) subunits increased Ccr4-Not association, suggesting these subunits normally limit Ccr4-Not interactions with Pol I (Fig. 6A and 6B). When Ccr4 was examined from cells lacking the A12.2 subunit, we also consistently detected that it migrated more slowly by SDS-PAGE relative to Ccr4 from wild-type or the other Pol I deletion mutants (Fig. 6A). The cause of this slower migrating form of Ccr4 is currently unknown.

To further delineate the functional relationship between Ccr4-Not and Pol I on rRNA expression, we combined a *ccr4Δ* with *rpa12Δ* and measured ETS1 and ITS1 rRNA synthesis in wild-type, the individual and double mutants cultured in nutrient rich media. Consistent with the results from Fig. 2B, *ccr4Δ* increased expression of both ETS1 and ITS1, while *rpa12Δ* modestly reduced ITS1 expression (Fig. 6C). The *ccr4Δ rpa12Δ* significantly reduced expression of ETS1 relative to both wild-type and *ccr4Δ* cells while marginally (*p = 0*.*047*, Student’s t-test) affecting ETS1 expression compared to *rpa12Δ* alone. Interestingly, while the *ccr4Δ rpa12Δ* reduced both ETS1 and ITS1 expression relative to *ccr4Δ* alone, *ccr4Δ rpa12Δ* restored the decreased ITS1 expression caused by the *rpa12Δ* mutation (Fig. 6C). This result is most likely explained by the decreased co-transcriptional rRNA processing caused by reduced SSU processome rDNA binding occurring in *ccr4Δ* cells (Fig. 3C). To determine the consequences these mutations have on Pol I rDNA binding, Pol I ChIP was performed. Consistent with the effects on ETS1 and ITS1 expression, *ccr4Δ* resulted in significantly increased Pol I rDNA binding compared to both wild-type and *rpa12Δ* (Fig. 6D). While the levels of rDNA bound Pol I in *rpa12Δ* relative to *ccr4Δ rpa12Δ* were not different, the double mutant significantly reduced Pol I binding compared to *ccr4Δ* (Fig. 6D). These results are also consistent with the reduced ETS1 and ITS1 expression detected in *ccr4Δ rpa12Δ* compared to *ccr4Δ* (Fig. 6C). Analysis of the 18S/SPT15 ratios from the *ccr4Δ*, *rpa12Δ* and *ccr4Δ rpa12* mutants show an increase in total rDNA copy number relative to wild-type, suggesting that all three mutants destabilize the rDNA (Fig. 6E). Importantly, the decrease in Pol I rDNA binding in the *ccr4Δ rpa12Δ* compared to *ccr4Δ* cannot be explained simply by a changes in rDNA copy number as the 18S/SPT15 ratios between these strains are not different. However, the 18S/SPT15 ratio is increased in *ccr4Δ rpa12Δ* relative to *rpa12Δ* alone, suggesting that impairment of both Ccr4-Not and the Pol I holoenzyme simultaneously enhances rDNA instability (Fig. 6E). These data suggest that Rpa12 may normally limit Ccr4-Not association with the Pol I holoenzyme and that the absence of this Pol I subunit during transcription may be compensated for by increasing interactions between Ccr4-Not and Pol I.

Although Rpa12 is genetically linked to Pol I elongation [24], we did not detect a significant defect in Pol I distribution in the *rpa12Δ* background compared to wild-type (Fig. 6D). As these experiments were performed under steady-state conditions, underlying defects in Pol I elongation are likely masked. Therefore, we analyzed further the impact of the *ccr4Δ*, *rpa12Δ* and *ccr4Δ rpa12Δ* mutants on Pol I elongation by assaying their growth on media containing the transcription elongation inhibitor 6-azauracil (6AU). Because 6AU depletes nucleotide pools, mutants deficient in either Pol I or Pol II elongation become hypersensitive to this compound while wild-type cells eventually adapt by upregulating IMP dehydrogenase expression [26,56]. Previous studies have demonstrated that Ccr4-Not mutants exhibit 6AU sensitivity which has been attributed to the function of Ccr4-Not as a Pol II elongation factor [57]. Consistent with these previous reports, *ccr4Δ* is already 6AU sensitive in the presence of 15 μg/mL 6AU (Fig. 6F). While Rpa12 promotes Pol I elongation in vivo [24], the *rpa12Δ* mutant does not exhibit 6AU sensitivity under these conditions which suggests that redundant elongation factors exist in vivo to compensate for Rpa12 loss. Surprisingly, the *ccr4Δ rpa12Δ* mutant exhibits a mild synthetic sick phenotype on control media and was completely growth impaired in the presence of 6AU (Fig. 6F). We next ascertained the consequences of these mutations on Pol I rDNA binding in vivo. These strains were cultured in nutrient rich media and an acute 6AU treatment, utilizing the same 6AU concentration (15 μg/mL) as above, was performed for 20 minutes before cross-linking and performing α-Pol I ChIP. Under these conditions, Pol I rDNA binding in wild-type cells was reduced to background across the entire rDNA locus, demonstrating that acute 6AU treatment temporarily prevents transcription reinitiation after the clearance of Pol I complexes already engaged in elongation (Fig. 6G). However, the *ccr4Δ*, *rpa12Δ* and *ccr4Δ rpa12Δ* mutants all retained detectable Pol I throughout the rDNA which indicates a reduced ability of Pol I to elongate through the rDNA. Surprisingly, *rpa12Δ* exhibited significantly lower Pol I levels relative to *ccr4Δ*, thus suggesting loss of Ccr4-Not function has a more severe effect on Pol I elongation (Fig. 6G). While we detected significant synthetic 6AU sensitivity in the *ccr4Δ rpa12Δ* relative to either *ccr4Δ* or *rpa12Δ* (Fig. 6G), this enhanced phenotype cannot be explained solely by effects on Pol I elongation as there was not increased Pol I retention in *ccr4Δ rpa12Δ* compared to the individual mutants. These data suggest that Ccr4-Not and Rpa12 likely function in at least partially parallel pathways to regulate Pol I elongation. This role for Ccr4-Not would be consistent with previous high throughput genetic analyses demonstrating synthetic sick and/or lethal phenotypes when Ccr4-Not mutants are paired with deletions of the non-essential Pol I subunits [58,59].

The above experiments demonstrate a role for Ccr4-Not in regulating both Pol I transcription initiation and elongation and suggest that Ccr4-Not is required for mTORC1-dependent Pol I regulation. To further delineate how Ccr4-Not functions in mTORC1-dependent Pol I regulation, we spotted wild-type, *ccr4Δ*, *rpa12Δ* and *ccr4Δ rpa12Δ* cultures to control plates or to plates containing low levels of the mTORC1 inhibitors rapamycin (3 nM) or caffeine (8 mM) to reduce mTORC1 signaling and mimic nutrient stress. These same cultures were also spotted to plates containing the DNA replication inhibitor hydroxyurea (100 mM) as well as to control plates that were incubated at 37°C to assay their sensitivity to heat stress. After three days growth, the effects of the various stress conditions on the individual mutants were assessed. While the *ccr4Δ* exhibited severe sensitivity to even modest mTORC1 inhibition or DNA replication stress, the *rpa12Δ* was not sensitive to any of these agents (Fig. 6H). Surprisingly, while mTORC1 inhibitors profoundly suppressed growth of *ccr4Δ*, the *ccr4Δ rpa12Δ* rescued this phenotype (Fig. 6H). This effect was not due to a simple increase in their general stress resistance as *ccr4Δ rpa12Δ* did not grow better than *ccr4Δ* in the presence of DNA replication stress, and the *ccr4Δ rpa12Δ* was actually ore sensitive to heat stress (Fig. 6H). Combined with the results from above demonstrating that the *ccr4Δ rpa12Δ* inhibits the increase in Pol I transcription that occurs in a *ccr4Δ* (Fig. 6H), these data support the conclusion that the severe sensitivity of Ccr4-Not mutants to mTORC1 inhibitors is largely due to deregulation of Pol I transcription.

## Discussion

The Ccr4-Not complex is an established Pol II transcriptional regulator that acts to regulate both transcription initiation and directly stimulate Pol II elongation. Whether Ccr4-Not also controls transcription by other RNA polymerases is currently unknown. In this report, we provide evidence that Ccr4-Not does indeed contribute to the control of Pol I transcription at both the initiation and elongation stages. In support, we demonstrate that Ccr4-Not physically associates with the rDNA and the Pol I holoenzyme and that cells deficient in Ccr4-Not function increase Pol I transcription specifically under nutrient rich conditions. The increased rRNA synthesis in Ccr4-Not mutants is specific to loss of Ccr4-Not function. These effects are not attributable to decreased rRNA processing or rRNA turnover mediated by RNA processing activites associated with Ccr4-Not, including Pan2-Pan3, TRAMP or the exosome as cells deficient in these activities do not increase rRNA levels. Instead, these Pol I transcriptional effects correlate with specific changes to core factor function and increased Rrn3-Pol I complex formation that occur only when Ccr4-Not mutants are grown in nutrient rich media. Ccr4-Not seems to limit Rrn3-Pol I complex formation in part by controlling the expression and/or turnover of Rrn3 specifically under these conditions which may normally restrict the number of initiation-competent Pol I complexes. Whether Ccr4-Not also impacts other aspects of Rrn3-Pol I regulation, such as their phosphorylation state which might modulate the strength and/or amount of Rrn3-Pol I complex formation, is unknown at this time. How Ccr4-Not disruption affects core factor is currently unclear. Although speculative, one possibility could be that Ccr4-Not disruption either increases core factor promoter binding and/or stabilizes its association with the promoter. Alternatively, physical remodeling of the core factor complex may occur in Ccr4-Not mutants such that the Rrn6 subunit becomes more accessible which might enhance its interactions with Rrn3. Since no increase in UAF or Rrn3 promoter association occurred in the Ccr4-Not mutant, we believe that an increase in the number of rDNA promoters bound by Rrn6, and hence an increase in the number of transcriptionally active rDNA repeats, is unlikely to explain these effects but cannot be completely ruled out at this time. Delineating these mechanisms in greater detail will be the subject of future studies. As Rrn6 contacts Rrn3 to facilitate transcription initiation, and formation of Rrn3-Pol complexes is a rate-limiting step for Pol I initiation, the changes to both core factor and Rrn3-Pol I complex formation in Ccr4-Not deficient cells could account for the increase in Pol I transcription [13–15].

Surprisingly, our data demonstrate that Ccr4-Not deficiency does not increase Rrn3-Pol I interactions or rRNA expression in cells grown under nutrient limiting conditions, suggesting that the role of Ccr4-Not in Pol I transcription is modified by the cellular nutrient environment. Ccr4-Not is functionally connected to both the Ras/PKA and mTORC1 pathways suggesting it could be a significant downstream effector of nutrient signaling [45,55]. However, these pathways and nutrient signaling in general, do not appear to affect Ccr4-Not interactions with Pol I since neither mTORC1 inhibition nor decreased Ras/PKA signaling altered their association. Since mTORC1 inhibition specifically causes Pol I nucleolar delocalization [46], these data suggest Ccr4-Not stably associates with Pol I even when Pol I is not bound to the rDNA or under conditions that reduce the rate of cell growth and proliferation. We also provide evidence that Ccr4-Not is required for mTORC1 to effectively reduce Rrn3-Pol I complex formation, and hence suppress Pol I transcription, when signaling through mTORC1 is repressed. Ccr4-Not likely contributes to this process by regulating expression or stability of Rrn3. Adjusting Rrn3-Pol I complex formation is an essential regulatory node for cells to bridge the rate of rRNA synthesis, and ultimately ribosome biogenesis, with changes in the nutrient environment. Therefore, disrupting Ccr4-Not uncouples Pol I transcriptional control from this key regulatory circuitry and likely explains part of its functional role in the mTORC1 signaling pathway.

Cells lacking functional Ccr4-Not are also deficient in rDNA binding by two other key transcriptional regulators, specifically the SSU processome and Hmo1. Decreased SSU processome binding would be predicted to contribute to some of the increase in rRNA containing ITS1 since SSU processome-dependent co-transcriptional cleavage would be impaired. However, the increase in ETS1 expression alone supports the conclusion that rRNA synthesis is elevated in Ccr4-Not mutants, a conlusion which is further reinforced by the increased binding of Pol I detected. More importantly, since SSU processome also promotes Pol I transcription, how increased Pol I transcription can be rationalized with decreased SSU processome binding is an intriguing issue. The simplest explanation is that the increased rate of Pol I transcription occurring may perturb and/or dislodge some of the SSU processome bound to the rDNA as it is a large molecular complex [60]. Likewise, the minor reduction in promoter binding by Hmo1 could be explained by the significantly increased number of initiating Pol I complexes which might in turn destabilize promoter bound architectural factors such as Hmo1.

Besides negatively regulating Pol I transcription initiation, we provide evidence that Ccr4-Not promotes Pol I transcription elongation as well. Ccr4-Not association with Pol I is modulated by the non-essential Pol I subunits such that Rpa34 promotes, while Rpa12 and Rpa14 oppose, Ccr4-Not interactions with Pol I. A recent study demonstrated that the non-essential Pol II subunits Rpb4 and Rpb7, which regulate Pol II elongation, are required for Ccr4-Not to interact with Pol II [61]. This study, combined with the results presented here, suggest that Ccr4-Not may as a general principle utilize non-essential polymerase subunits as a means for modulating its interaction with RNA polymerase complexes. These interactions are functionally relevant to Pol I transcriptional control since individual Ccr4-Not or Rpa12 mutants reduce Pol I elongation in vivo while the combinatorial mutant exhibits a synthetic sick phenotype and dramatically enhanced sensitivity to the transcription elongation inhibitor 6AU. These data suggest that Ccr4-Not and Rpa12 function in at least partially parallel pathways to promote Pol I elongation. However, although the *ccr4Δ rpa12Δ* mutant significantly opposed the increased Pol I transcription caused by *ccr4Δ* alone, the double mutant did not enhance Pol I retention on the rDNA when challenged with 6AU. The inability to detect enhanced Pol I elongation defects occurring in *ccr4Δ rpa12Δ* is at least partially explained by the simultaneous increase in Pol I transcription initiation occurring in Ccr4-Not deficient cells which is likely confounding the analysis of Pol I elongation in vivo. Future studies will require careful dissection of the role Ccr4-Not plays in initiation versus elongation to gain a complete understanding of how Ccr4-Not acts at these two distinct stages of the Pol transcription cycle. Importantly, a role for Ccr4-Not as both a positive and negative regulator of Pol I transcription would be consistent with its still poorly understood functions in Pol II transcriptional regulation [35].

One of the most intriguing observations from our studies is that *ccr4Δ rpa12Δ* reduces the enhanced Pol I transcription occurring in *ccr4Δ*, yet it simultaneously rescues the extreme sensitivity to mTORC1 inhibition caused by *ccr4Δ*. Pol I transcription acts dominantly to coordinate expression of both Pol II and Pol III transcribed genes specifically involved in ribosome biogenesis [20,21]. Therefore, uncoupling Pol I control from upstream mTORC1 regulation through Ccr4-Not deficiency might disrupt the normal stoichiometry of ribosomal biogenesis and contribute to the induction of a stress state that is growth inhibitory. Previous studies support this concept. For example, uncoupling Pol I transcription from upstream mTORC1 signaling by direct fusion of Rrn3 to the A43 Pol I subunit enables cells to grow normally under nutrient rich conditions. However, when mTORC1 signaling is reduced, these cells are significantly growth impaired [20,21]. Additionally, overexpressing rRNA from a heterologous promoter in mTORC1 mutants, as well as mutants in chromatin pathways affecting Pol I transcription, also sensitizes these cells to mTORC1 inhibition [62].

Studies of Pol I transcriptional regulation have focused mostly on the initiation phase of the transcription cycle and have identified the promoter bound factors required for Pol I transcription initiation in both yeast and mammals. The importance of the ancillary factors involved in Pol I transcription, as well as the regulation of Pol I elongation, are emerging as important means for controlling rRNA biogenesis [4]. As such, Ccr4-Not appears to be an ancillary factor shared between the Pol I and Pol II transcription systems that is critical for bridging nutrient signaling through mTORC1 with Pol I transcriptional regulation and control of cell growth and proliferation.

## Materials and Methods

### Chemical reagents and plasmid construction

All chemical reagents utilized in this study, including rapamycin, caffeine, and 6AU were purchased from Fisher Scientific or Sigma-Aldrich. The *CCR4* and *ccr4-1* expression vectors were generated by utilizing plasmids pJCN100 (*CCR4*) and pJCN101 (*ccr4-1*) as templates in a PCR reaction with *CCR4* primers containing a C-terminal mono-FLAG sequence. The resulting PCR fragments were cloned into the Xba I and EcoRI sites of the p416ADH expression vector [63].

### Yeast strains and growth conditions

All yeast strains were derived from the BY4741 genetic background. Yeast strains and plasmids are listed in S1 Table. All yeast deletion mutants and genomically-tagged strains were generated utilizing PCR-generated targeting cassettes as previously described [64]. Antibiotics utilized for strain generation were purchased from Invitrogen or GoldBio. For yeast cells grown under nutrient rich conditions, the media used was 1% yeast extract, 2% peptone, and 2% dextrose (YPD). Cells grown under nutrient defined conditions were cultured in synthetic complete (SC) consisting of 0.17% yeast nitrogen base without amino acids, 1 g/L of glutamic acid, 2% dextrose, and 1.9% drop-out mix with the appropriate drop-out nutrients added back. SC media reagents were purchased from US Biologicals. To select for yeast cells carrying plasmids, cells were grown in SC dropout media lacking either uracil or leucine. Cells were cultured to mid-log phase (OD_600_ = 1.0–1.5) at 30°C in either YPD or SC media for the experiments outlined in this study. All yeast spotting assays were performed utilizing cultures grown overnight in the appropriate growth media. Cell numbers were then normalized based on their OD_600_ reading, five-fold serially diluted and spotted to the appropriate plate media. Cells were typically incubated for three to five days at 30°C unless otherwise specifically stated. For 6-azauracil assays, the indicated strains were transformed with a *URA3* containing plasmid and cultured overnight in SC-ura media before spotting to either control SC-ura media or SC-ura media containing 15 μg/mL 6-azauracil. Plates were then incubated for six days at 30°C.

### ChIP and qPCR analysis

Yeast strains were cultured in either YPD or the appropriate SC media to an OD_600_ = 1.0–1.5 before cross-linking with 1% formaldehyde for 15 minutes followed by a five minute quench with 125 mM glycine at room temperature with gentle shaking. ChIP extracts and immunoprecipitations were performed exactly as described previously utilizing 400–500 μg of ChIP extract [62]. 10% of total ChIP extract was also purified as the input control. Purified IP DNA was eluted in 50 μL of qPCR quality water and then diluted 1:5 while the input DNA was eluted in 100 μL and subsequently diluted 1:50. Purified DNA was analyzed by qPCR on an Applied Biosystems StepOne Plus real-time PCR machine utilizing rDNA specific primers and the data were normalized with the formula 2^(Input Ct-IP Ct)^ and expressed as % IP/Input. Primer sequences are listed in S2 Table.

### Total RNA isolation and gene expression analysis

Total RNA was isolated by resuspending yeast pellets in Tri-Reagent (Sigma-Aldrich) in the presence of chloroform and glass beads. Samples were homogenized by vortexing and then centrifuged for two minutes at maximum speed. The soluble fraction was removed, precipitated with isopropyl alcohol, and centrifuged for 15 minutes at 4°C to pellet the nucleic acids. Pellets were resuspended in 50 μL of DEPC-treated dH_2_O in the presence of DNase I and digested for 30 minutes at 37°C. Samples were then phenol/chloroform extracted, precipitated, and resuspended in 50 μL DEPC-dH_2_O. For cDNA synthesis with the Im-Prom II cDNA synthesis system (Promega), 1 μg of total RNA was utilized with random hexamer primers, per the manufacturer’s instructions, in a total volume of 20 μL. After synthesis, purified cDNA were diluted to a final volume of 100 μL and then 1:5 dilutions were utilized for qPCR analysis with gene-specific primers listed in S2 Table. qPCR analysis was performed as described above and the gene-specific signals were normalized to the expression of the *SPT15* housekeeping gene using the formula 2^(SPT15 Ct- Target gene Ct)^. Wild-type was set to a value of 1 while all yeast mutants were expressed relative to wild-type. All results were presented as the average and standard deviation of four or more independent experiments and the statistical significance determined by Student’s t-test.

### Protein analyses and antibodies

Yeast whole-cell extracts were prepared as previously described and 500 μg of extract was utilized for immunoprecipitations in extract buffer (300 mM NaCl, 10 mM Tris pH 8.0, 0.1% NP-40, and 10% glycerol) containing protease and phosphatase inhibitors [62]. Immune complexes were isolated using Protein A-conjugated agarose beads and washed extensively, resolved by SDS-PAGE, transferred to PVDF membrane, and immunoblotted with the appropriate antibody. Ubiquitin-conjugated Rrn3 was analyzed using a similar approach as previously described [36]. Wild-type and *not4Δ* cells expressing Rrn3-1XMyc/7X-histidine were cultured as described above in YPD, and then cell pellets were weighed and equivalent amounts of pellet were lysed in denaturing buffer (8M urea, 300 mM NaCl, 50 mM Tris pH 8.0, and 0.5% Triton X-100) by bead beating at 4°C for six minutes in the presence of glass beads. Cell lysates were clarified by centrifugation at 15,000 rpm at 4°C for 15 minutes. From the clarified supernatant, 50 μL were removed and used as the total extract. The remainder of the supernatant was rotated with 50 μL of Ni^2+^-conjugated agarose beads at room temperature for two hours followed, by three washes in denaturing buffer before resolving samples by 8% SDS-PAGE and immunoblotting. All immunoblots were developed utilizing Immobilon ECL reagent (Millipore) and exposure to film. Quantification of immunoblots was performed using ImageJ. Antibodies used for immunoblot and ChIP analyses were as follows: α-HA (mouse, Santa Cruz), α-Myc (mouse clone 4A6, Millipore), α-β-actin (Abcam), α-ubiquitin (mouse, Santa Cruz), α-G6PDH (rabbit, Sigma-Aldrich) and general IgG (rabbit, Santa Cruz).

## Supporting Information

S1 Table Plasmids and yeast strains utilized in this study. (DOCX)

S2 Table PCR primers utilized in qPCR analyses. (DOCX)

S1 Supplemental References Supplemental references. (DOCX)
