# Supplementary material for: Ccr4-Not Regulates RNA Polymerase I Transcription and Couples Nutrient Signaling to the Control of Ribosomal RNA Biogenesis
Source: PLoS Genet. 2015 Mar 27;11(3):e1005113. doi: 10.1371/journal.pgen.1005113 (PMC4376722; doi:10.1371/journal.pgen.1005113)
Supplement: S1 Table — (DOCX) [file pgen.1005113.s001.docx]

**S1 Table. Plasmids and yeast strains utilized in this study.**

| Plasmid | Description | Reference |
| --- | --- | --- |
| p416ADH | *Amp^R^ CEN6/ARSH4 URA3 ADH1*prom/CYC1term |  |
| p*CCR4* | Amp^R^ *CEN6/ARSH4 URA3* *ADH1*prom-*CCR4*-*FLAG/ CYC1*term | This study |
| p*ccr4-1* | *Amp^R^* *CEN6/ARSH4 URA3* *ADH1* prom-*ccr4-1*-*FLAG/ CYC1* term | This study |
| pRS425 | *LEU2* 2μ | [1] |
| pPHY1299 | pRS425; *PDE2* | [2] |
|  |  |  |
| Yeast Strain | Genotype | Reference |
| BY4741 | MATa his3Δ1 leu2Δ met15Δ ura3Δ0 | Open Biosystems |
| *ccr4Δ* | BY4741; *ccr4::KanMX* | Open Biosystems |
| *not4Δ* | BY4741; *not4::KanMX* | Open Biosystems |
| *caf1Δ* | BY4741; *caf1::KanMX* | Open Biosystems |
| *caf40Δ* | BY4741; *caf40::KanMX* | Open Biosystems |
| *caf130Δ* | BY4741; *caf130::KanMX* | Open Biosystems |
| *not3Δ* | BY4741; *not3::KanMX* | Open Biosystems |
| *not5Δ* | BY4741; *not5::KanMX* | Open Biosystems |
| *trf4Δ* | BY4741; *trf4::KanMX* | Open Biosystems |
| *rrp6Δ* | BY4741; *rrp6::KanMX* | Open Biosystems |
| *pan2Δ* | BY4741; *pan2::KanMX* | Open Biosystems |
| *rpa12Δ* | BY4741; *rpa12::KanMX* | Open Biosystems |
| *rpa14Δ* | BY4741; *rpa14::KanMX* | Open Biosystems |
| *rpa34Δ* | BY4741; *rpa34::KanMX* | Open Biosystems |
| *rpa49Δ* | BY4741; *rpa49::KanMX* | Open Biosystems |
| Ccr4-TAP | BY4741; *CCR4-TAP::HIS3* | Open Biosystems |
| Not1-TAP | BY4741; *NOT1-TAP::HIS3* | Open Biosystems |
| Not2-TAP | BY4741; *NOT2-TAP::HIS3* | Open Biosystems |
| H3239 | BY4741; *CCR4::MYC13::HIS3 MX6* | [3] |
| NOY886 | *MAT_ rpa135_::LEU2 ade2-1 ura3-1 his3-11 trp1-1 leu2-3,112 can1-100 fob1_::HIS3 pNOY117 (CEN RPA135 TRP1)   rDNA repeat~ 42* | [4] |
| NOY1051 | *MAT_ rpa135_::LEU2 ade2-1 ura3-1 his3-11 trp1-1 leu2-3,112 can1-100 fob1_::HIS3 pNOY117 (CEN RPA135 TRP1)  rDNA repeat~143* | [4] |
| YNL318 | BY4741; *RPA190-6XHA::HphNT1* | This study |
| YNL320 | YNL318; *RPA190-6XHA::HphNT1 ccr4::KanMX* | This study |
| YNL358 | H3239; *CCR4::MYC13::HIS3 MX6 RPA190-6XHA::HphNT1* | This study |
| YNL325 | BY4741; *UTP9-6XHA::HphNT1* | This study |
| YNL399 | BY4741; *ccr4::KanMX UTP9-6XHA::HphNT1* | This study |
| YNL459 | YNL318; *HMO1-9XMYC::NatMX4* | This study |
| YNL461 | YNL320; *HMO1-9XMYC::NatMX4* | This study |
| YNL494 | *CCR4-9XMYC::NatMX4 RPA190-6XHA::HphNT1* *rpa12::KanMX* | This study |
| YNL495 | *CCR4-9XMYC::NatMX4 RPA190-6XHA::HphNT1* *rpa49::KanMX* | This study |
| YNL496 | *CCR4-9XMYC::NatMX4 RPA190-6XHA::HphNT1* *rpa34::KanMX* | This study |
| YNL499 | *CCR4-9XMYC::NatMX4 RPA190-6XHA::HphNT1* *rpa14::KanMX* | This study |
| YNL506 | YNL320; *RRN5-9XMYC::NatMX4* | This study |
| YNL508 | YNL318; *RRN5-9XMYC::NatMX4* | This study |
| YNL504 | YNL320; *RRN3-9XMYC::NatMX4* | This study |
| YNL510 | YNL318; *RRN3-9XMYC::NatMX4* | This study |
| YNL526 | *rpa12::KanMX ccr4::NatMX4* | This study |
| YNL608 | *RPA190-6XHA:: HphNT1 RRN6-9XMYC:: NatMX4* | This study |
| YNL609 | *RPA190-6XHA:: HphNT1 RRN6-9XMYC:: NatMX4 ccr4::KanMX* | This study |
