# Supplementary material for: Ccr4-Not Regulates RNA Polymerase I Transcription and Couples Nutrient Signaling to the Control of Ribosomal RNA Biogenesis
Source: PLoS Genet. 2015 Mar 27;11(3):e1005113. doi: 10.1371/journal.pgen.1005113 (PMC4376722; doi:10.1371/journal.pgen.1005113)
Supplement: S2 Table — (DOCX) [file pgen.1005113.s002.docx]

| Primer | Sequence | Reference |
| --- | --- | --- |
| rDNA prom forward | TTCCGTATTTTCCGCTTCC | This study |
| rDNA prom forward | TTCTTTTGCCCTCTCTGTCG | This study |
| ETS1 forward | TGGGTTGATGCGTATTGAGA | This study |
| ETS1 reverse | TCGCTGATTTGAGAGGAGGT | This study |
| 18S forward | GCTTGCGTTGATTACGTCCC | [1] |
| 18S reverse | CACTAAGCCATTCAATCGGT | [1] |
| ITS1 forward | TGTTTTGGCAAGAGCATGAG | This study |
| ITS1 reverse | TCGAATGCCCAAAGAAAAAG | This study |
| 25S forward | CGTTCATAGCGACATTGCTT | [1] |
| 25S reverse | GGGTGAACAATCCAACGCTT | [1] |
| Term forward | GAGAAAAGCTCATTTCCTATAGTTAACAG | [2] |
| Term reverse | TTCACTTGTCTCTTACATCTTTCTTGG | [2] |
| 5S forward | GCGGCCATATCTACCAGAAA | This study |
| 5S reverse | CTGAGTTTCGCGTATGGTCA | This study |
| qHMO1 forward | GGAAGCAGGCTTACAACGTC | This study |
| qHMO1 reverse | TTTTCCACAGGAGGTTCAGC | This study |
| qRRN3 forward | CAGCCGACGGTAGTCAAAGT | This study |
| qRRN3 reverse | TCACTTGCTCTTCCACATGC | This study |
| qRRN5 forward | CCTTTTTCTGGTGCCTTTCA | This study |
| qRRN5 reverse | CACTCAGCGCTCATTTCGTA | This study |
| qSPT15 forward | TAAAAAGAGCTGCCCCAGAA | This study |
| qSPT15 reverse | ATGATGACAGCAGCAAAACG | This study |

**S2 Table. PCR primers utilized in qPCR analyses.**
