# Supplementary material for: Ccr4-Not Regulates RNA Polymerase I Transcription and Couples Nutrient Signaling to the Control of Ribosomal RNA Biogenesis
Source: PLoS Genet. 2015 Mar 27;11(3):e1005113. doi: 10.1371/journal.pgen.1005113 (PMC4376722; doi:10.1371/journal.pgen.1005113)
Supplement: S1 Supplemental References — (DOCX) [file pgen.1005113.s003.docx]

**Supplemental References**

1. Brachmann CB, Davies A, Cost GJ, Caputo E, Li J, et al. (1998) Designer deletion strains derived from Saccharomyces cerevisiae S288C: a useful set of strains and plasmids for PCR-mediated gene disruption and other applications. Yeast 14: 115-132.

2. Howard SC, Hester A, Herman PK (2003) The Ras/PKA signaling pathway may control RNA polymerase II elongation via the Spt4p/Spt5p complex in Saccharomyces cerevisiae. Genetics 165: 1059-1070.

3. Swanson MJ, Qiu H, Sumibcay L, Krueger A, Kim SJ, et al. (2003) A multiplicity of coactivators is required by Gcn4p at individual promoters in vivo. Mol Cell Biol 23: 2800-2820.

4. French SL, Osheim YN, Cioci F, Nomura M, Beyer AL (2003) In exponentially growing Saccharomyces cerevisiae cells, rRNA synthesis is determined by the summed RNA polymerase I loading rate rather than by the number of active genes. Mol Cell Biol 23: 1558-1568.

5. Hontz RD, Niederer RO, Johnson JM, Smith JS (2009) Genetic identification of factors that modulate ribosomal DNA transcription in Saccharomyces cerevisiae. Genetics 182: 105-119.

6. Reiter A, Hamperl S, Seitz H, Merkl P, Perez-Fernandez J, et al. The Reb1-homologue Ydr026c/Nsi1 is required for efficient RNA polymerase I termination in yeast. EMBO J 31: 3480-3493.
